# APHASIA: HOW OUR LANGUAGE SYSTEM CAN “BREAK”

**DOI:** 10.3389/frym.2022.626477

**Published:** 2022-04-04

**Authors:** Maria V. Ivanova, Nina F. Dronkers

**Affiliations:** 1 Aphasia Recovery Lab, Department of Psychology, University of California, Berkeley, Berkeley, CA, United States; 2 Department of Neurology, University of California, Davis, Davis, CA, United States

## Abstract

Our brains enable us to learn language. We develop it early on in life and use it effortlessly every day. It is only when the language system breaks down that we fully realize how complicated it is to speak and understand. In this article, we will explore what happens when brain damage leads to a language disorder called aphasia. About 15 million people worldwide and about 2 million in the U.S. alone are affected by aphasia. Sadly, many people still do not know what aphasia is. Here, we will explain different types of aphasia, tell you about the language difficulties people with this disorder encounter, and provide information about how language is processed in the brain.

## Graphical Abstract

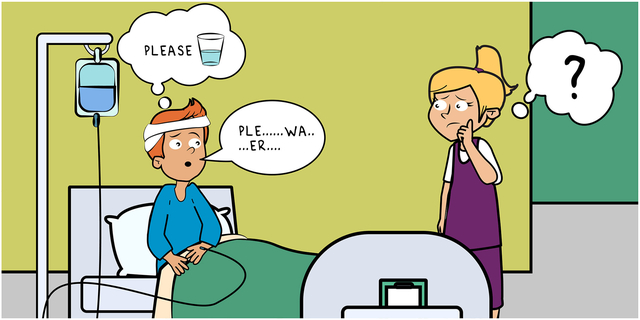

Can you describe what is happening in the picture shown in Figure 1? Without much effort, you can probably put together a simple storyline, turning your ideas into words, and those words into neat sentences, much like beads on a thread. Communication is essential for human beings; it has played a crucial role in the evolution of our species and remains an important part of our society. Language is the most common, efficient, and informative means of communication, whether it is by exchanging day-to-day stories with our friends, learning in the classroom, reading the texts of ancient scholars, or focusing on one’s thoughts and feelings during a solitary walk in the park. Our brains enable us to learn language, we develop it early on in life and use it effortlessly every day. The complex nature of language only becomes apparent when the system breaks down and we cannot make sense of what is being said to us or find the right words to express our thoughts.

**LANGUAGE**

A system of spoken, manual (signed), and/or written symbols (words and expressions) that are used and understood by a large group of people to communicate.

## WHAT IS APHASIA?

The brain is the organ that makes language possible. Various regions of the brain work together to process and understand incoming speech and to respond by turning our thoughts into words, combining them into phrases, and then producing clear, meaningful sounds that people around us can easily understand. The brain needs a lot of energy to do its demanding job well. About 20% of the calories that we consume daily go directly to fuel the brain, even though the brain is only 3% of the body’s weight. The brain also needs a constant supply of oxygen, delivered via the blood, to work properly. Brain cells with reduced or no oxygen supply can survive for a limited time, but if blood flow is not restored quickly, they start to die. If blood cannot reach areas of the brain that are responsible for language, and the brain cells there suffer permanent damage, a person can lose the ability to use language normally and is said to have an aphasia (the Ancient Greek word for “speechlessness”).

**APHASIA**

Is a language disorder caused by an injury to the brain. In aphasia, the ability to talk, to understand spoken language, to read and write are all impaired, to varying degrees.

Aphasia is a communication disorder in which a person’s ability to talk, to understand spoken language, and to read and write will all be affected, to varying degrees. These language impairments lead to communication problems. Aphasia is not caused by poor hearing or vision problems, or difficulty moving the tongue or jaw. A person is diagnosed with aphasia when there is observable damage to the brain, as seen on a brain scan (learn more about brain scans in another FYM article [1]). Stroke is the leading cause of aphasia in adults. A stroke can happen either when a blood vessel becomes blocked and cannot deliver oxygen to brain areas down the line, or when a vessel breaks, leading to blood spilling out into the brain. You can find out more about stroke in another FYM article [2]. Aphasia can also be caused by a traumatic brain injury, surgery on the brain, or an infection in the brain.

**IMPAIRMENT/DEFICIT**

Difficulty or loss of ability to perform a certain function or task. For aphasia, the impairment or deficit is a loss of language skills that happened because of the injury.

**STROKE**

When a blood vessel in the brain gets clogged or breaks and causes the blood to stop flowing leading to brain tissue damage.

About 15 million people worldwide and about 2 million in the U.S. alone are affected by aphasia. Roughly 35% of those who have a stroke will end up with aphasia. Sadly, many people still do not know what aphasia is. A survey conducted by the National Aphasia Association in 2016 showed that <10% of the U.S. population has heard about aphasia and can identify it as a language disorder. This makes social participation particularly challenging for people with aphasia, as many people they encounter will not understand the disorder and the person with aphasia will probably find it difficult to explain the condition.

## IS APHASIA THE SAME FOR EVERYONE?

The language impairments observed in aphasia can be strikingly different in each person. Some people with aphasia will find it more difficult to understand others, while others will make sound errors and grammatical mistakes while speaking. Still others will have difficulty repeating words and sentences. However, certain features of aphasia are almost universal.

All people with aphasia have problems with retrieving words in a conversation. However, depending on where the damage is in the brain, this common deficit will manifest itself in different ways. Someone might confuse the right word with related words, like choosing “letter” when meaning to say “envelope.” Someone else might struggle to retrieve the word, sort of like when you speak in a foreign language and are limited in your vocabulary. For example, when asked to name a picture of an Egyptian pyramid, a person with aphasia said, “Can not think of the word, it is from Hawaii, not Hawaii, but that part of the world, where someone was buried, I can see it, but can not name it.” Yet another person will make errors in the sounds of the words and say “bee” instead of “key.” Observing and describing these different error patterns has greatly expanded our understanding of how the language system works and how many distinct brain areas are required to support the simple act of producing a single word.

**Figure 1:**
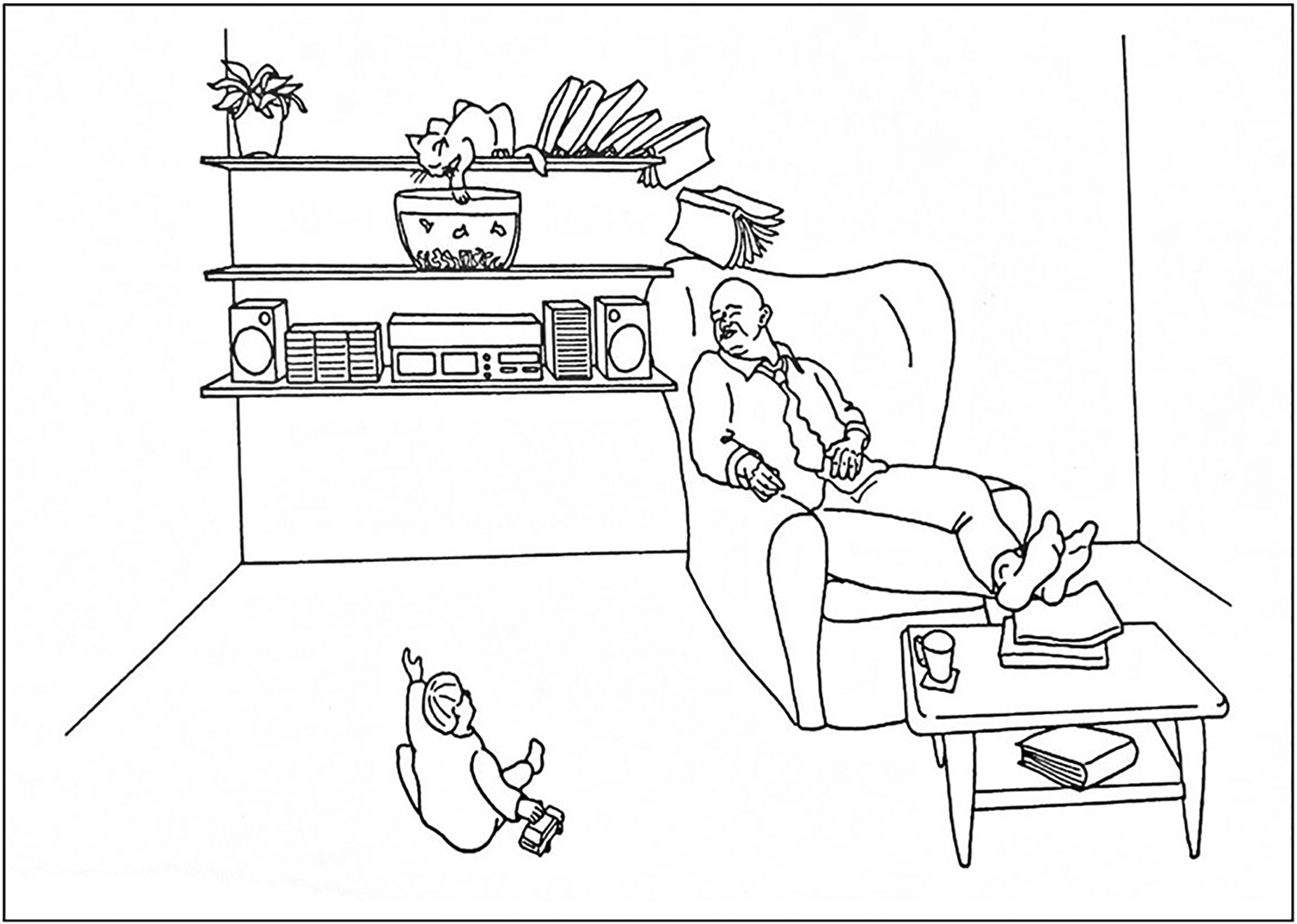
Can you describe what is going on in this picture? Image from the Comprehensive Aphasia Test [3]. Reprinted with permission.

**Figure 2:**
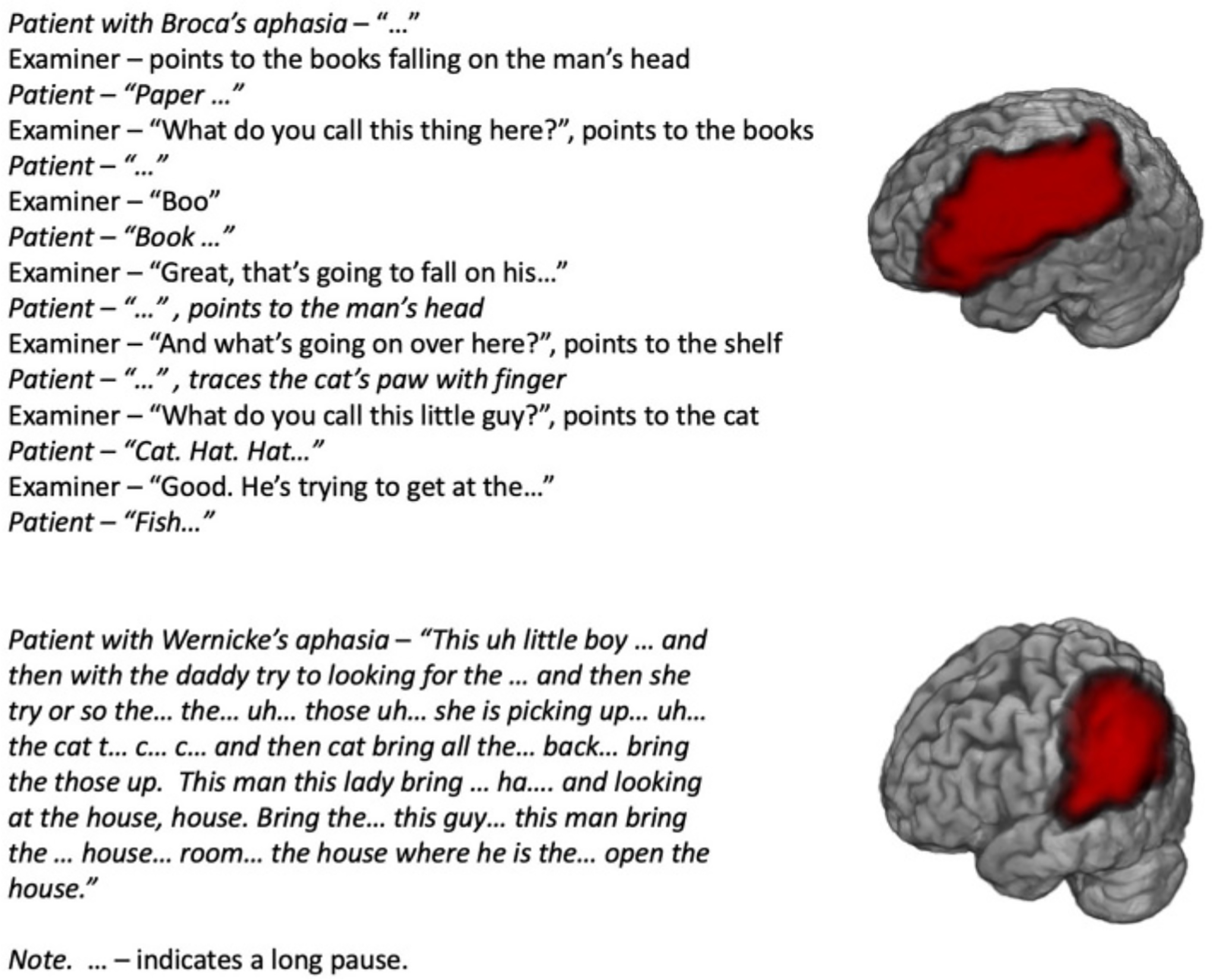
Descriptions of the picture in Figure 1, provided by two patients with different (Broca’s and Wernicke’s) types of aphasia. Their damaged brain areas are shown in red on the brain images. Damage to different brain areas causes aphasia to manifest itself in different ways. As you can see, some patients have a lot of trouble coming up with even common words, while others talk more effortlessly, though it might be hard to understand them.

To accurately describe the characteristics of aphasia, researchers have devised numerous language tests, in which patients must match words and sentences to pictures, name pictures, repeat words and phrases, read, and write. Remember the picture in Figure 1? It comes from a commonly used aphasia test—the Comprehensive Aphasia Test [3]. This picture description task can tell us a lot about aphasia (Figure 2).

## TYPES OF APHASIAS

**Figure 3:**
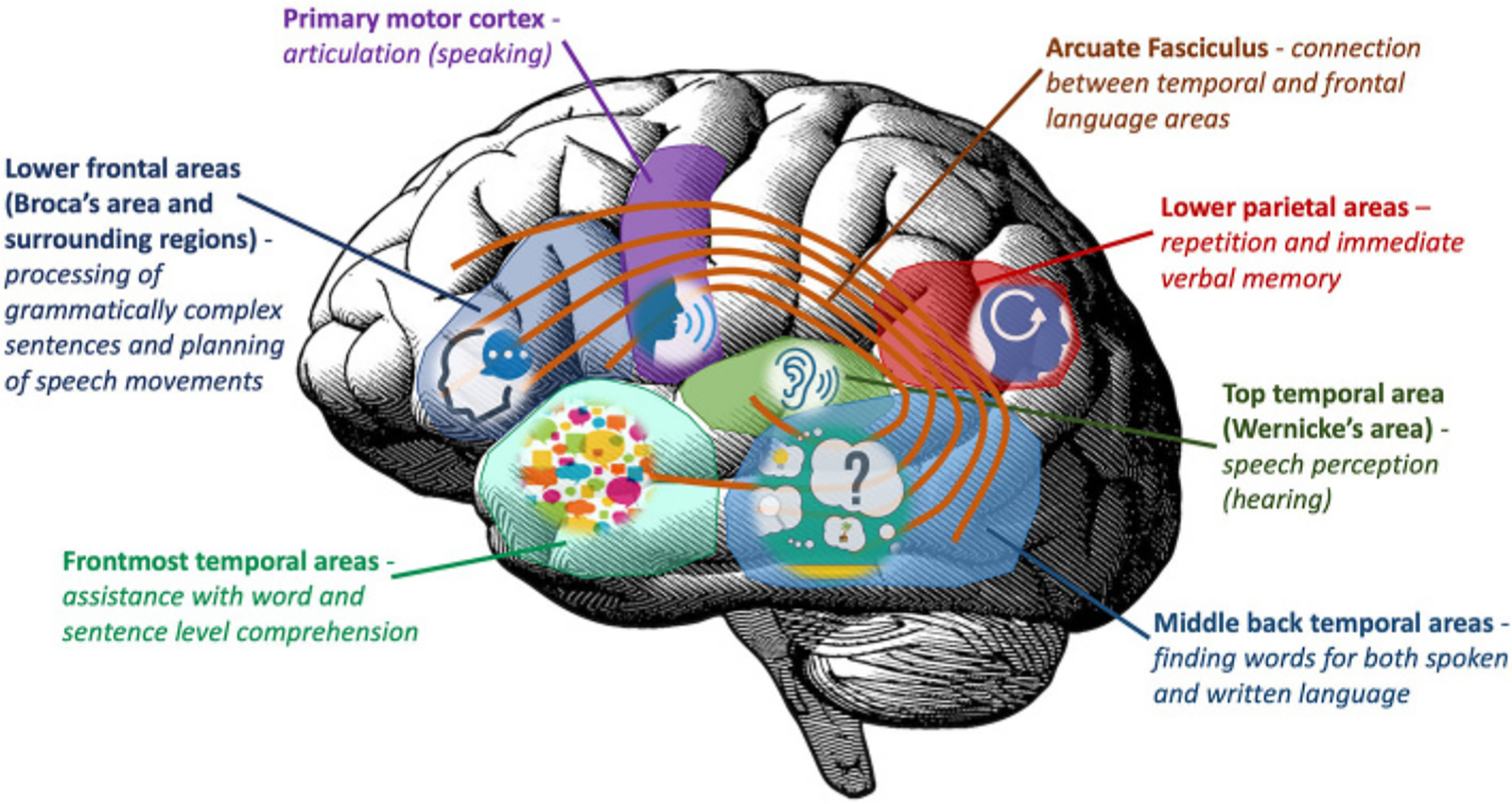
Incoming speech is first processed in the top temporal area (Wernicke’s area). Then the meaning of the words is accessed in the middle back temporal areas, along with selection of an appropriate response. Next, through connections called the arcuate fasciculus, information is sent to the lower frontal language areas (Broca’s area and surrounding regions). There, the sentence is put together and the response is spoken with the help of the primary motor cortex. Additional areas are recruited depending on the type and difficulty of the language task. Damage to one or more of the language areas can lead to aphasia.

Even the first doctors describing aphasias in the 1800s were aware of the varied nature of the disorder. However, they quickly recognized that certain symptoms tend to appear together, thus a classification system for aphasia was born. Originally, it was thought that language depends on two critical brain regions—one in the temporal lobe (Wernicke’s area) for understanding language and one in the frontal lobe (Broca’s area) for speaking. However, we know today that the language system depends on multiple brain regions working together in a coordinated fashion (Figure 3). Most aphasias result from damage to the left hemisphere (side) of the brain, since it is the left hemisphere that most often processes language. There are many types of aphasia; the most well-known are explained below.

**WERNICKE’S AREA**

The upper back part of the left temporal lobe that was believed to be important for understanding language. Currently, researchers think that a much larger area is responsible for understanding language.

**BROCA’S AREA**

The lower part of the left frontal lobe that was thought to be responsible for language production. Today, scientists believe it is involved in word selection and some grammatical processing.

Broca’s aphasia was the first to be described, by a French surgeon, Paul Broca, in 1861. The most obvious symptom in this aphasia is trouble saying words and sentences. In very severe cases, when damage to the areas responsible for language production is extensive, the patients’ speech will be limited to 1–2 words or even just a string of sounds or a meaningless phrase that they repeat in response to any question. In milder cases, individuals tend to produce grammatical errors in their speech, for instance, leaving out proper verb endings and using simplified phrases that contain only the most important content words. People with Broca’s aphasia also have problems with comprehension, especially with complex sentences, like, “Put the comb on the other side of the pen and turn over the book.” Typically, Broca’s aphasia is caused by injury to the lower part of the left frontal lobe of the brain (called Broca’s area) and nearby brain structures.

Wernicke’s aphasia has a pattern of symptoms that is almost the opposite of Broca’s aphasia, with comprehension as the main problem. First described in 1874 by a German doctor, Carl Wernicke, it is characterized by severe comprehension difficulties combined with well-pronounced and easy speech that often does not make much sense. Sometimes the person cannot even understand the simplest sentences, like, “Point to the door.” They may talk a lot, but often choose the wrong words or produce meaningless sounds. Their problem seems to be in matching words to the concepts they want to describe or understand. Originally, it was thought that damage to a part of the temporal area (called Wernicke’s area) led to Wernicke’s aphasia. Today researchers have established that damage to a much larger part of the left temporal lobe causes lasting Wernicke’s aphasia.

In conduction aphasia, the most common symptom is a problem with repetition, especially for words that do not have a real meaning, like “blosh” or meaningless phrases like “waltzing private island.” People with conduction aphasia can speak quite well and understand most of what is being said to them but repeating a phrase word-for-word is very difficult. They often say, “I heard you say it, but the words just disappeared!” Typically, Conduction aphasia is associated with damage to lower parietal and top temporal areas.

Global aphasia is the most severe form of aphasia, in which a person has the symptoms of all the aphasias we have just described. They have a lot of trouble talking and understand very little. Yet, they can often communicate through facial expressions and gestures, and can still “think,” though not with words. Many enjoy working on puzzles, listening to music, being around other people, and traveling with their families or friends. Being without language is hard, but other forms of communication and non-verbal activities can still provide meaningful social interactions.

Kids can get aphasia too, but childhood aphasia is rare. Strokes can happen in children born with a heart condition or a blood disorder that leads to an interruption of normal blood flow to the brain. Most kids will show good recovery, but some might have language problems for the remainder of their lives.

## HELPING PEOPLE WITH APHASIA

While most people with aphasia can regain a substantial part of their language, this can take many years after a stroke, and most people say their language never fully recovers. For almost 150 years, researchers have been trying to understand how the brain processes language, how language can be lost due to brain damage, and how to best help patients recover their language skills. Research with people who have aphasia has provided valuable insights about the disorder itself and the language system, in general. This research has led to the development of language therapies for people with aphasia. Trained speech-language pathologists work with individuals with aphasia to help them relearn lost words, understand complex sentences, and regain the ability to produce words accurately. Some people benefit more from therapy than others, and researchers still do not fully understand why. Today, doctors are also testing whether stimulating brain regions with mild electrical current or magnetic waves might help brain cells be more active and form new connections. Thanks to research, we know that it is beneficial to start therapy as soon as possible and engage the whole family in helping the person regain their language skills.

Even when recovery of language skills is not fully possible, it is important to continue to provide people with aphasia with opportunities for meaningful social participation, so that they do not feel isolated or depressed. If someone you know has aphasia, speak slowly, and use simple sentences and words. Make sure there are no additional distractions in your environment and only one person is talking at a time. Give the person with aphasia more time to respond and listen patiently. Be creative in your communication—use writing, gestures, and/or pictures to facilitate understanding. Remember that, while the person with aphasia has lost a part of their language, they have not lost their personality or intelligence. There are many resources you can use to learn more about aphasia and how to communicate with those who have it.^1^

Many mysteries about language and the brain still remain. It will take research by kids like you to fully understand how language works and how we can best help people with aphasia. Acquiring and using language seems easy, but losing it is painful and regaining language ability is difficult. So, the next time you talk to someone, pause for a second and appreciate the enormous complexity of our language system and yet how naturally it makes us who we are. Value this fragile human ability and be ready to give a helping hand to those who are challenged every time they must communicate with another person.

## Footnotes

1 The American Speech-Language-Hearing Association: https://www.asha.org/public/, The National Aphasia Association: https://www.aphasia.org/, The National Institute on Deafness and Communication Disorders: https://www.nidcd.nih.gov/health/aphasia, Aphasia Recovery Lab at the University of California Berkeley: https://aphasia.berkeley.edu/resources-for-patients/
